# Self-Configuration and Self-Optimization Process in Heterogeneous Wireless Networks

**DOI:** 10.3390/s110100425

**Published:** 2010-12-31

**Authors:** Lucas Guardalben, Luis Javier García Villalba, Fábio Buiati, João Bosco Mangueira Sobral, Eduardo Camponogara

**Affiliations:** 1 Computer Science Program, Distributed Mobile Computing—Network Security (DMC-NS) Group, Federal University of Santa Catarina—UFSC, Florianópolis, SC, Brazil; 2 Group of Analysis, Security and Systems (GASS), Department of Software Engineering and Artificial Intelligence (DISIA), School of Computer Science, Office 431, Universidad Complutense de Madrid (UCM), Calle Profesor José Garía Santesmases s/n, 28040 Madrid, Spain; 3 System and Automation Department, Federal University of Santa Catarina—UFSC, Florianópolis, SC, Brazil, E-Mails: guardalben@inf.ufsc.br (L.G.); fabio@fdi.ucm.es (F.B.); bosco@inf.ufsc.br (J.B.); camponog@das.ufsc.br (E.C.)

**Keywords:** WMN, self-organization, OLSR, AODV

## Abstract

Self-organization in Wireless Mesh Networks (WMN) is an emergent research area, which is becoming important due to the increasing number of nodes in a network. Consequently, the manual configuration of nodes is either impossible or highly costly. So it is desirable for the nodes to be able to configure themselves. In this paper, we propose an alternative architecture for self-organization of WMN based on Optimized Link State Routing Protocol (OLSR) and the ad hoc on demand distance vector (AODV) routing protocols as well as using the technology of software agents. We argue that the proposed self-optimization and self-configuration modules increase the throughput of network, reduces delay transmission and network load, decreases the traffic of HELLO messages according to network’s scalability. By simulation analysis, we conclude that the self-optimization and self-configuration mechanisms can significantly improve the performance of OLSR and AODV protocols in comparison to the baseline protocols analyzed.

## 1. Introduction

WMN communicate through multi-hops and they consist basically of client-nodes and mesh-routers. The first one is able to give support to the mobility and the second one are fixed nodes. Both types of nodes possess wireless interfaces for communication among them, and the mesh-routers are responsible for forming the mesh network, in an autonomous or pre-determined way. The mesh-routers can work as gateways or bridges, allowing the interconnection of different types of networks [1]. The mesh-routers are usually placed in the roof of buildings to avoid electromagnetic interference in the range of their antennas. WMN are an emerging technology and it possesses advantages related to maximize their covering area. This is due to the possibility of introducing mesh-routers, with the gateway functionality, connected in a similar way to the wired network, by extending the applications for the WMN and also their low cost implementation because they can use wireless technologies available in the market. The WMN are a good alternative for projects in large-scale wireless networks [2–4].

Usually the WMN are deployed in metropolitan areas and they consist of a large number of nodes. The main advantages of the WMN are fault tolerance, configuration simplicity, and large bandwidth capacity [5]. In contrast to other networks—as is the case of cellular telephony—where the failure of a gateway in a single base-station (BS) compromises communication, in case of fail, the networks remain very communicable. WMN provide high fault tolerance even if several nodes fail. According to [6], a wireless mesh network works in any communication network that has a partial topology or that is completely linked. Particularly, these networks are characterized by wireless static nodes which support a distributed infrastructure for the client-nodes on a topology in partial mesh. Although WMN and the ad hoc ones are similar, the protocols and architectures available for the latter are not easily applied to the former. For instance, if a wireless mesh network uses multiple radio interfaces, the routing protocol initially designed for mobile ad hoc networks should be adapted to work with multiple-radios, as well as the routing metrics should be improved.

**Figure 1.**
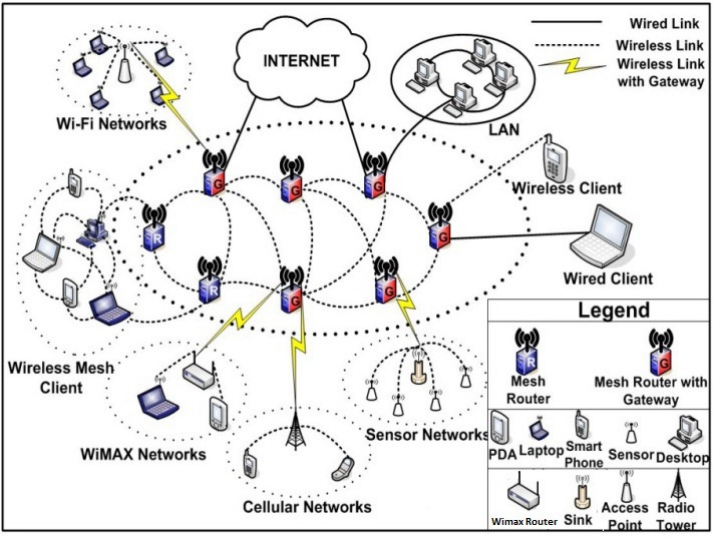
Hybrid WMNs.

An example of hybrid mesh network is shown in Figure 1. This architecture is the combination between the infrastructure and the client meshing, which the mesh clients can access the network through mesh routers as well as directly meshing with other mesh clients.

In WMN, it is desirable that the routing protocols minimize the overhead of messages and avoid a high throughput of messages for discovering routes in the network. Most of the protocols for these networks derived from the ad hoc networks and they need to be adapted to the characteristics of the WMN. Because there is great mobility among nodes in mobile ad hoc networks, whereas in the case of the wireless mesh the mobility is limited, a routing protocol specifically designed for the mobile and ad hoc networks will not deliver maximum performance in a wireless mesh network. Due to this limitation there is a need of new routing protocols or adaptations of the existing protocols for mobile ad hoc networks in order to maximize the performance of WMN [7–11]. Besides the limitation of the routing protocols, another desirable characteristic for WMN is the self-organization capacity (self-organization), which is the organization of the network without manual intervention of the administrator system [12].

This paper presents an approach for self-organization of WMN by using the software agent technology [13] for information acquisition jointly with the autonomic computation modules known as self-x, which are: self-configuration, self-optimization, self-healing, and self-protection. The modules developed in this research are limited to the self-configuration. As a first experience, we used the routing protocol OLSR and AODV to validate the proposed approach, whereby exhaustive simulations with different scenarios were performed using the OpNET simulator (Optimized Network Engineering Tools).

This paper is organized as follows: Section 2 introduces some related work and motivation for research. Section 3 contains an overview of the architecture for self-organization. Section 4 presents the experimental results and Section 5 discusses the results analyzed for the self-optimization and self-configuration process. Finally, Section 6 contains our conclusions and suggestions for future work.

## 2. Self-Organization in WMN: Related Works

Due to the increasing number of devices and services, efforts are being made so that network installation, administration, and maintenance do not become unmanageable to the administrator. Therefore, research works have addressed these issues.

In [14], a project for a community wireless mesh self-organized network is presented. This work presents a case-study for multi hop networks, showing how limitations can be overcome and how wireless devices affect mesh performance.

Raniwala *et al.* [15] present a multi-channel wireless mesh network architecture (called Hyacinth) that equips each mesh network node with multiple 802.11 network interface cards (NICs). The authors show that the intelligent channel assignment is critical to Hyacinth’s performance, present distributed algorithms that use only local traffic load information to dynamically assign channels and to route packets, and compare their performance against a centralized algorithm that performs the same functions through a simulation study.

In [16], a project to adopt a model for autonomic computing is presented, which shows how self-managing autonomic capabilities can be achieved in an evolutionary manner. Further, it describes initiatives for industry standards that are necessary to carry out these applications of autonomic computing within an open system architecture for heterogeneous environments.

Vandenberghe *et al.* [17] have proposed a architecture system which is able to deal with the limited and often conflicting requirements to build wireless automation. This architecture is based on management separation, control and data plan. The authors also identify the different components and functions needed for communications over heterogeneous wireless networks with end to end QoS support.

The architecture of the wireless mesh network presented in [18] called MobiMESH, has been implemented in a real life testbed designed with high mobility support and with integration capabilities. Mobility management is supported by a set of procedures that build an intermediate stratum between layer 2 and layer 3. Experimental results and performances are shown and problems and future works are outlined.

In [19], the authors present a system denominated SCOMAN (Self-Configuring and Optimizing MANET’s), which implements self-configuration and self-optimization for ad hoc networks.

The authors propose a collaborative administration module for MANET’s based on administration policies, which was implemented using the concepts of *context awareness* and *cross layer design*. It can be noticed that the proposed approach is only applicable to ad hoc networks and will need adaptations to use in WMN.

In [20], a wireless mesh network is considered as a candidate to solve the last mile problem in high speed network services. Traditional mesh networks face the challenge of scalability due to the features of self-organization and multi-hop connection. To this aim, this author has proposed a hierarchical architecture for large scale mesh networks. This kind of network is divided into small sub-networks which operated independently and are connected through a reliable backbone network. The proposed architecture addresses routing and security issues. Self-organization is a promising subject for the next generation of wireless networks, but approaches that support self-organization of such networks are still lacking.

### 2.1. Others Related Management Projects

In this subsection, some autonomous management projects are explained.

#### ANA: Autonomic network Architecture

This is an initiative established by European Foundation, that has some companies and universities as partners such as: ETH Zurich, NEC, University of Oslo, University of Waterloo, University of Basel, University of Lancaster, Fokus, University of Liege, Pierre University et Marie Curie, NKUA and Telecom Austria. It has as aim the development of a goal-architecture for autonomous networks [21].

The main aims of the project are:

- *Scientific:* In this aim concern to identify the principles of the autonomous computing, that allows the network could not only be scalable in size but also in functionalities. Therefore, some self-x attributes are desirable as auto-optimization, the self-monitoring, self-repair and self-protection.
- *Technological:* The technological aim concerns in constructing one *framework* that is able to interconnect different types of networks in an independent way and without direct intervention of the administrator of the network. The self-organization concept is developed considering each node individually inside the network.

The architecture allows the dynamic adaptation and reorganization of traffic according to the user’s necessity. Besides the dynamic adaptation, the architecture still supports mobile nodes and multiple administrative domains.

The architecture of each node and the architecture of the network. In the architecture of each individual node the application layer can be perceived, that provide services as www, email, ftp, DNS, to peer-to-peer (kazaa, emule) voip (Skype), among other supported application. The concept of compartment is also introduced in the architecture of a node. The concept of compartment is implemented according to operational rules and administrative politics for a given communication context. In which it defines as a node enters and leaves the network, who are the registered members, the authentication model and how the node is communicated with another member of the network (routing, communication *to peer-to-peer*, addressing). The compartment concept configures the system of communication in small units of management; this enables the network to have a local vision of each node, in order to provide a self-organization at global level. The process of self-association and organization has the function to associate the new fixed nodes or not, that want to enter in the network. This process has one high level of abstraction for the user. The architecture of the network has the property of discovering services or resources, in which they abstract a list of services that each node can provide; an example would be a node with bigger capacity of storage and another one with capacity for printing.

#### CASCADAS:Component ware for Autonomic, Situation-aware Communications and Dynamically Adaptable Service

This project was initiated in January 2006 and founded by *European Commission*, aiming development of architecture for self-organization and communication services. The CASCADAS project uses the model of *Autonomic Communication Element* - ACE. An ACE acts as entity, that can implement a distributed way of communication and services and also act as a central point of services [22]. In this case a ACE model must integrate all the self-organized capacities and must be the basis for the implementation of the situated services of communication in the application layer, as well as the basis of implementation for the network layer and the level layer of *middleware*. In Cascades project the application layer, distinguishes the dynamic development of components, with the coupling of components for autonomous applications. In the layer of *middleware* or *middle* we can see some capacities as self-cure (self-healing), pervasive supervision and knowledge of the network, that are dynamically aggregate functions according to the autonomous behavior of the network. In the network layer, we notice the integration of different devices as: personal television sets, personal computers and even sensors. Besides that, the question of sharing computational resources can also be noticed.

#### FOCALE: Autonomic Network Management Architecture

This is a distributed architecture, where each network element can incorporate the autonomic management functionalities, and the main requirements [23]:

- Accommodate legacy components
- Ensure that AEMCs (Autonomically Enabled Managed Component) can also be efficiently managed
- Provide a means to orchestrate the behavior of ACs (Autonomic Components)
- Ensure that all ACs adjust their functionality according to policy
- Provide a means to reason about the environment and recommend or take appropriate actions, so that the underlying business goals are not violated and, hopefully, optimized.

The main goal of FOCALE is provide a architecture that adapts to changes in the environment, business rules and user requirements.

The final, after analyzing the related works, we concluded that existing solutions [15,17–20,24] partially address the self-organization problem, thereby encouraging us to work towards self-organization by defining an alternative architecture which provides a first experience in low cost of the main capacities, namely: self-configuration, self-optimization in WMN.

## 3. An Architecture for Self-Organization

**Figure 2.**
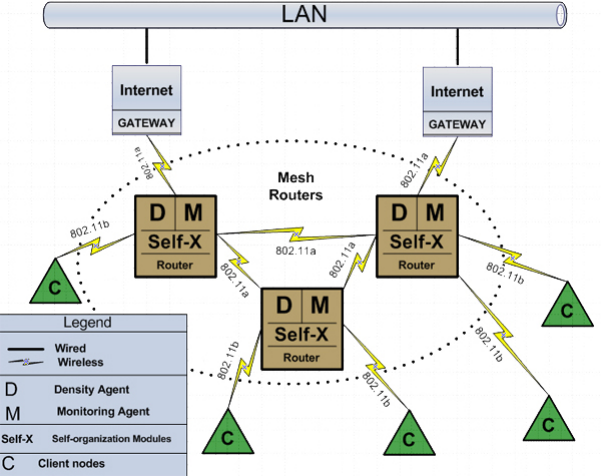
General vision of the self-organization model proposed, adapted to the networks in WMN.

The architecture for self-organization in WMN was projected to adapt to the context of the gateway nodes. The Figure 2 shows the use of the self-organization architecture in a typical scenario. The gateway nodes are connected to the wired Ethernet network providing access for the Internet to mesh routers. The mesh routers, forms the backbone of network using the 802.11 standard. The communication between the client nodes and mesh routers are made through the standard 802.11 g. This process is classified as single radios, because the backbone and the connection among clients’ nodes are made by using the same network interface card (NIC). In the agents’ layer two types of agents were developed and these are: NS (*Network-Size*) and NM (*Network-Monitoring*). In the self-organization process was integrate the capacity of Self-X (configuration, optimization, healing and protection) in each node-gateway in mesh. The clients’ nodes are associated with the mesh router, according to the covering area. The definition of WMN can be represented through an unidirectional graph G = (R,L), where R is the group of mesh routes, and L is the group of connections between two nodes gateways x,y. Some nodes x can maintain a connection with the node *gateway* g and can be connected to the Internet.

Each client node can establish bi-directional connections with node gateway x, as it can be observed in the Figure 2.

The clients nodes can be so much mobile, as fixed and they differ from the type of the device: class of mobile devices (PDA’s, cellular telephones and laptops) and class of fixed devices (computers desktop, servants and node-gateways).

**Figure 3.**
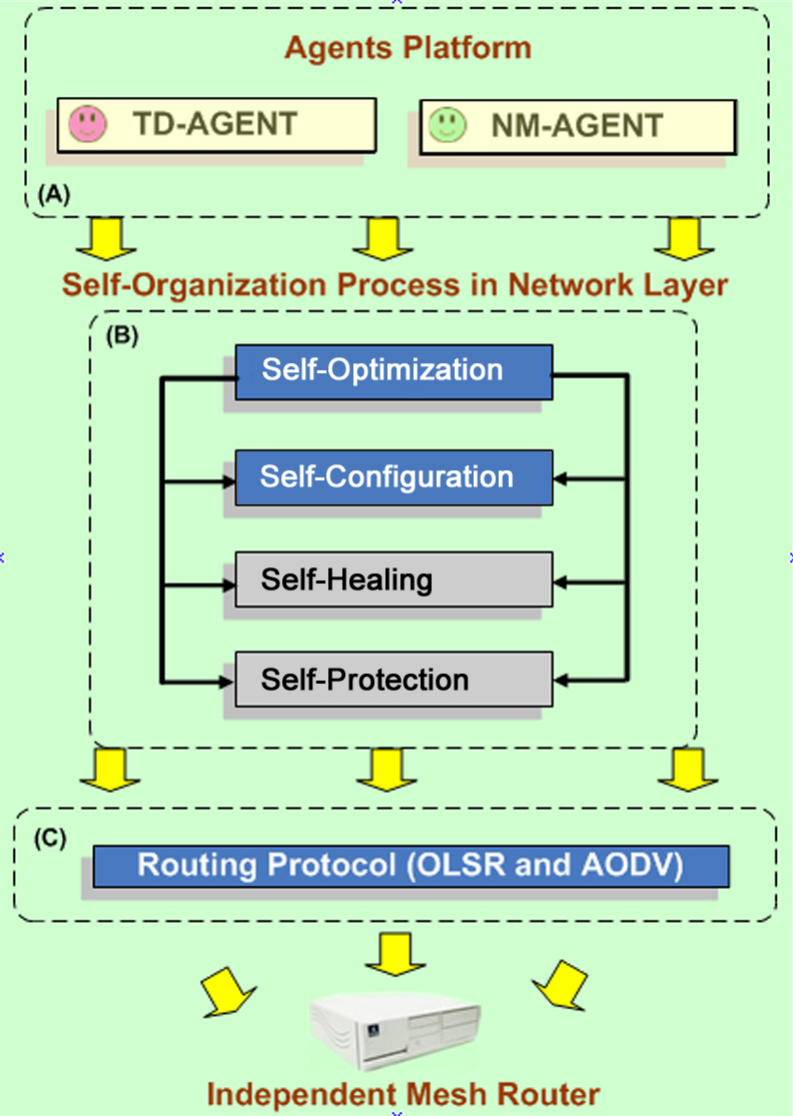
Vision of the architecture for Self-Organization for a node-gateway in mesh.

For a better explanation about the logic interaction among the modules involved in this work, it grew the architecture based in layers. The approaches based on layers guarantee flexibility and simplicity in the development, in the sense that all of the involved functions are well defined and treated in an independent way. This is an advantage regarding the detection of isolated flaws, as it can be seen in the Figure 3. In this same Figure it is observed that the self-cure capacity and self-protection that are in tones of dark ash, they were not implemented in the mark of this work, they were only implemented together self-optimization capacity and self-configuration with NS_Agent.

The details of each layer are described in the following sections and they stand out in a conceptual way in the interaction among all of the proposed layers.

### 3.1. Agents Platform

With the intention of doing the monitoring and control of the network, software agents were used [13] for the administration of the network’s size and also of the monitoring of the nodes gateways. The software agents are considered an alternative to the conventional architecture Client/Server, differing from the local interaction with code mobility [25–27].

### 3.2. Agents for Monitoring and Control of the Network’s Size

The monitoring is an important factor for the knowledge of the system to be managed. Being observed this importance, the software agents’ technology was proposed to do the monitoring of the nodes gateways inside the mesh of the network. Besides the monitoring, the agents are responsible to maintain the list of the network’s nodes updated, in other words, the number of equipments in the network connected in certain periods of time.

That information is relevant so that the self-configuration process through the size of the network, it can configure the parameters of the routing protocols in a dynamic way. It chose to use software agents due to the characteristics found in this approach type such as a fault tolerance (has abilities to react dynamically to adverse situations), autonomous interaction and asynchronous, (it’s capable to hold in an autonomous way with other agents) and dynamic adaptation (Saint capable to adapt to changes in the atmosphere). Among a lot of characteristics found in the software agents’ behavior, those are the most desirable in the context of this work. Another important factor for the software agents’ choice as approach for distributed monitoring, is that approaches as SNMP possess as characteristics models centralized that they are not applied in WMN, leaving like this an only point of flaws, carting problems in the modules that need that functionality.

The agents that compose the layer application play a specific role in the architecture and are described to proceed:

- **(A) *TD-Agents*:** Mobile agents that are placed in the mesh router and in each association of the client node of a mesh router. The agent has specific functions in the discovery of the network density, which can vary between the small-scale, medium-scale, and large-scale. The values for scales (small, medium and large) are defined under [28]. A suite of *TD-Agents* forms the basis of **self configuration** capability of the AODV and OLSR protocols.
- **(B) *NM-Agents*:** Responsible for the monitoring of network behavior, including the packet loss rate, delay, signal range, throughput, latency, and active and inactive nodes, as well as information about the link state. They are fixed agents in the mesh routers, and facility the self-optimization capability in the AODV and OLSR protocols.

### 3.3. Self-Organization Processes

Self-organization arises in mesh wireless networks by embedding self-x abilities (optimization, configuration, cure, and protection) in the network routing protocol. Such self-x abilities render the routing protocol more autonomously, thereby increasing performance, fault tolerance, and security of the network. Details on the implementation of these abilities are given below, with emphasis on the self-optimization and self-configuration. It is worth remarking that the self-x abilities were implemented in the network layer as extensions to the standard services of the routing protocols OLSR and AODV.

### 3.4. Self-Optimization

The self-optimization module is responsible for selecting the parameters of the protocols AODV and OLSR that maximize network performance, while considering that WMN varies with the number of clients. The self-optimization module accounts for:

- the OLSR and AODV parameters that mostly influence network performance;
- the type of the parameters (integer, real or boolean); and
- the size of the network at the current time, which is obtained from the NS-Agent.

The first step is to model network performance as a function of the routing protocol parameters. The analysis of simulation data elicited that the hyperbolic cotangent [29] captures the average influence of the parameters on network performance. Thus, the function modeling the influence of a given parameter was obtained by solving a linear least squares problem to fit the function below to simulation data:

(1) $$\mathrm{FO}(x)=\left(\frac{e^{2x}+1}{e^{2x}-1}\right)$$

The solution was in this case, the use of non-linear optimization without restrictions, so all the features of the problem fit themselves in this optimization sub-area.

In the self-optimization module of the parameters we note that it should be found a minimum local that could replace the standard values of the protocols’ parameters OLSR and AODV in the size scales proposed as: small, medium or large.

(2) $$\mathrm{Minimize}\sum_{i=1}^{n}\left[\mathrm{FO}(i)-y(i)\right]$$

In Equation (3) it was used to minimize the objective function (1). Where *y* is the result of the throughput analysis for each proposed interval of time.

### 3.5. Self-Configuration

This module is responsible for setting the parameters of the routing protocols OLSR and AODV based on the information available to the NS-Agent. The parameters are defined to improve the computation of routes in the network.

The chief factors influencing the parameters are: the size of the network, the mobility rate, the transmission distance, the strength of the transmission signal, and the network overhead, jitter, and delay. The system administrator has to assist the configuration of the parameters to avoid parameter set-ups that compromise the network performance, specially so in complex networks. Typically, the system administrator does not tune the parameters for lack of knowledge or time, selecting default values that in many situations influence network performance drastically and potentially create security breaches. Therefore, it is desirable to have a self-configuration module for obvious reasons: reduced intervention from the part of the system administrator and consequently a reduction in installation and maintenance costs. Self-configuration relieves the system administrator from these tedious tasks who can concentrate his efforts on more critical decisions. To that end, Algorithm.1 has been designed to configure the network parameters based on three different network sizes: small, medium, and large. That is, the self-configuration module selects a suit of routing protocol parameters that have been optimized by the self-optimization module to improve network performance depending on the size of the network. The identification of the network size is based on the information received from the NS-Agent, this way allowing the network to gracefully adapt to the prevailing conditions as new clients arrive.

The idea behind the algorithm is to set the routing protocol parameters for OLSR and AODV to the predefined values depending on the network size. Therefore, the algorithm maintains a list of the client nodes currently connected to the network, which can be obtained from the NS-Agent at any given instant. Once the self-configuration module is up and running, the system administrator not longer needs to configure routing protocol parameters manually to optimize network performance in response to dynamic changes in network size (*i.e.*, number of client nodes).

The boundaries between the three network sizes were defined according to works in the literature [5,28]. Namely, a network is said to be small if it has from 1 to 30 nodes, medium if it has more than 30 but not more than 70 nodes, and large if it has more than 70 and does not exceed 100 nodes. Evidently, the algorithm described above can be augmented with other policies for self-configuration that improve network performance at particular locations of the network.

### 3.6. Self-Cure and Self-Protection

The self-cure module is responsible for detecting, localizing, and repairing faults in the routing protocols OLSR and AODV: when a router halts or stops working properly, the remaining router nodes should detect the fault with the help of the monitoring agent (MN) and create alternative routes for the connections that have been compromised by the faulty router. On the other hand, the self-protection modules should enforce security within the network, possibly using techniques based on immunological systems [30–32] in an effort to detect intrusion in complex WMN.

A feature of the architecture proposed in this paper is the potential to use existing routing protocols for wireless networks, such as OLSR and AODV. Further the architecture can be extended to operate with other routing protocols, which would require adjustments in the self-x modules to support a given protocol. With all its functionalities implemented, the architecture would suppress all the limitations of existing routing protocols for WMN with respect to performance, fault tolerance, and security.

As a first experience, we proposed modules for self-optimization and self-configuration with focus on the WMN performance of the network. Several simulation experiments were performed to assess the effectiveness of the proposed architecture and the self-x modules that were implemented. The conceptual design of the architecture was proposed in [33], while the implementation of the self-configuration module for OLSR was presented in [34].

**Algorithm. 1:**
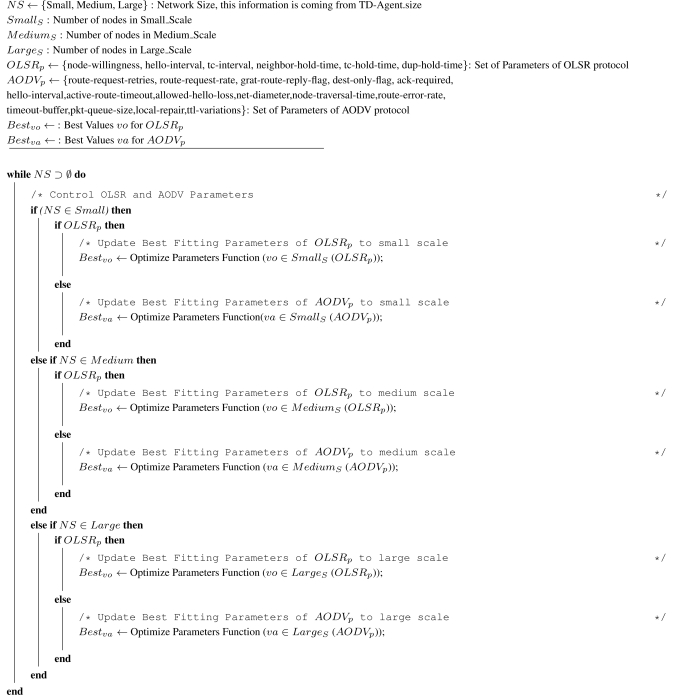
Control Scales and Parameters of OLSR and AODV.

All in all, the proposed architecture can be thought of as an extension of existing routing protocols. The motivation to this research was not the development of a self-organizable routing protocol from scratch, but rather the extension of the concepts of self-organization to existing routing protocols for wireless networks.

## 4. Experiments and Results

In this section it will be presented the experimental results of this paper.

### 4.1. Process Models Developing

The process models were developed with the purpose to simulate and to validate the architecture in a specific way, in other words, each self-organization capacity suggested together with the change of messages used by the agents’ platform.

To simulate the proposed architecture it developed the process modules in language *C/C*++ and it was used as sequence of cycles FSM (*Finite State Machines*), in other words, machines of finite states to organize sequence of steps of each module of developed process. The simulation tool OPNET (*Optimized Network Engineering Tool*) *Modeler*(www.opnet.com) supports for mobile networks and structure to simulate networks in wide climbs. To compile the process modules the Visual compiler C++ version 6.0 was used and later it changed in an automatic way for the simulator *OPNET*, in files in the EMU format (*External Modulates Application*), which are files interpreted by the simulation tool.

The models of processes presented in the following sections were developed in layers, beginning for the implementation of the agents’ platform, later for the self-organization process (being limited to the self-optimization capacities and self-configuration) and finally applying those capacities as extension for the routing protocols OLSR and AODV.

### 4.2. Model of Process Agents

In this model, software agents were considered as static in each node gateway. Each agent is coupled inside the node-gateway. For the platform’s development it didn’t grow all of a software agent’s functionalities as it can be seen in [13], because that was not the mark of this work. it grew in the simulator the change of messages among the agents. The NS-Agents were divided in NS-Agent (it Sends), that it has the specific function of sending messages for NS-Agent (it Receives), with all of the information about the amount of devices contained in the network. That is made by message change that is better detailed in the subsection below.

The model of the node gateway in the simulator OPNET contains all of the functions of a gateway, in other words, from of the access to the physical layers (Wireless_Lan_Mac), of network, of transport to the application layer.

For the message exchange among the agents grew up a format of specific package containing the following fields (source address, destination address, amount of mesh routers nodes, amount of clients nodes, amount of gateway nodes and total of all nodes in the network). Each node-gateway have these fields locally. Later these fields are encapsulated in a package that is sent of times in times for all of the neighboring nodes of a certain gateway, with the intention of obtaining the list of the size of the network always updated.

In function of the method used for sending of these packages to be flooding, a time of sending of the updating of the list should be configured, that can vary according to the accumulation of messages in the network. The configured standard time is measured in seconds, therefore to every 50 seconds the gateways-nodes send their information, avoiding like this the accumulation of exchange of the agents’ messages.

The NM-Agents are also divided in NM-Agent (it Sends) and NM-Agent (it Receives) and they have the function of monitoring the nodes gateways in mesh that they are active or not in the network. The NM-Agent (it Sends) has the function of contacting NM-Agent (it Receives) of times in times. That process form the self-cure base proposed for this dissertation, however that capacity still was not implemented in this work.

### 4.3. Models of Process of the Capacities of Self-Optimization and Self-Configuration

The Self-optimization capacities and Self-configuration have a strong correlation, in other words, the self-configuration method is complemented with the self-optimization method, while a capacity configures the parameters according to the network’s scale, the other optimizes the values to be coupled in the protocols’ parameters OLSR and AODV as an extension to the same ones.

To implement the model of self-optimization process, it modified the original modules of the protocols OLSR and AODV (aodv_rte.pr.c and olsr_rte.pr.c) that take part of the support for networks MANET of the simulator OPNET and it was added to the self-optimization function and self-configuration. Countless modifications were made in the structure of the modules with intention to adapt those capacities. The self-optimization method is responsible for the choice of the value optimized to be put in each parameter of the routing protocols OLSR and AODV. To obtain the optimized value, a network was simulated in WMN Intra-campus as the scenario presented in the Figure 5. In this scenario the variation of the amount of nodes clients was simulated and associated in each node gateway, together with the variation of the interval of the chosen parameters for this experiment.

**Figure 4.**
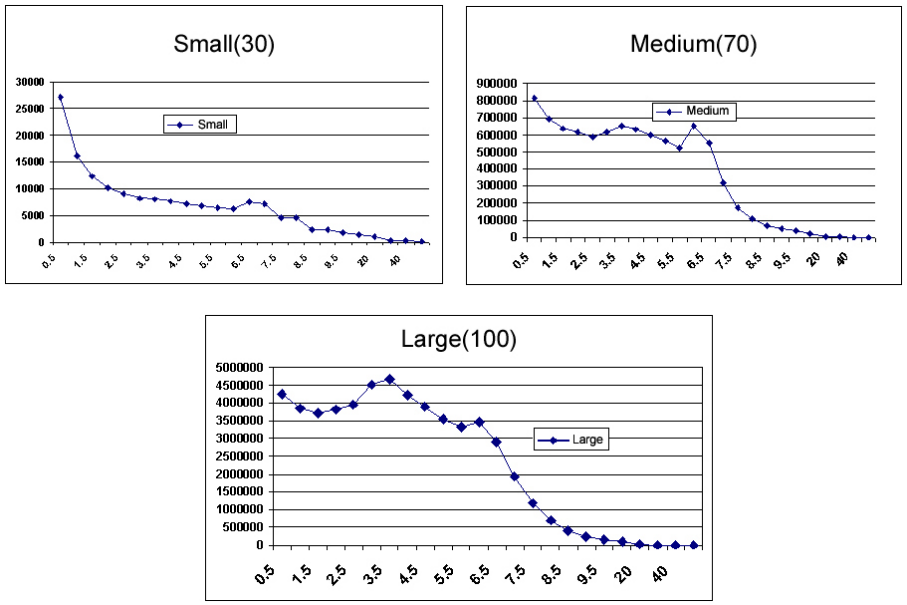
Example of the analysis of the scales of the parameter Hello-Interval according to the metric of throughput and the variation of the interval of time.

In Figure 4, an example of the curve behavior can be observed created through the relationship throughput versus variation of the time of messages Interval HELLO.

In this example we noticed that the analysis of the variation of parameter’s value was made in an independent way for the three proposed scales, small, medium and large. We can also see that the proposal objective function adapted from a satisfactory way to the graphs of the Figure 4, resulting in a optimized value for each proposed scale. Regarding to optimization process of the generated curve, we choose to use the software MATLAB with the optimization function *lsqnonlin*, this function is used to solve adjustments no linear problems, and it was used in this work to aid in the adjustment of the curve created by the specific analysis of each parameter. According to above mentioned in the Section 3.4, the hyperbolic, cotangent function adapt to the behavior of the generated curve. With the obtained data of the Table 1 the curve was drawn in the three scales and, after that, it was researched an objective function in the literature that was better adapted to the behavior of the generated curve. After tests and analysis in the simulator, the hyperbolic cotangent function was chosen, because this obtained the best behavior among the other objective functions analyzed (linear and tangent interpolation hyperbolic).

To exemplify the analysis process the parameter Hello-Interval was used as reference, but the same process was made for the other chosen parameters.

As shown in the Table 1 the example of the data can be seen as only obtained of the parameter Hello-Interval. Still in this example, it is noticed, that for the analysis of Hello-Interval the interval of time of 0.5 was varied up to 50 seconds and also the amount of node-clients that was varied the scale of the network accordingly: SMALL (0 to 30), MEDIUM (30 to 70) and LARGE (70 to 100).

In the Tables 2 and 3 the parameters of the protocols can be visualized OLSR and AODV after the self-optimization process. With intention to analyze the parameters in a specific way, five parameters of the protocol AODV were chosen (Route-Request-Retries, Hello-Interval, Active-Route-Timeout, Allowed-Hello-Loss, Net-Diameter) and five parameters of the protocol OLSR (Hello-Interval, Tc-Interval, Neighbor-Hold-team, Tc-Hold-team, Duplicate-Hold-team). This analysis was accomplished being varied the interval of time of each parameter and analyzing the growth of the network according to the scales. Therefore was used the throughput metric of the network.

Tables 2 and 3 they contain all of the parameters of the protocols OLSR and AODV with their respective parameters already optimized, with the self-optimization method. That table was coupled in the self-configuration method and later the results were abstracted.

Besides the specific analysis of the five parameters some other parameters were also modified to privilege the network according to the scale. As an example of that, the parameter Node-Willingness can be mentioned that takes part of the group of parameters of the protocol OLSR. In this parameter it was configured for *medium* in the small scale, *high* for medium scale and *always* for large scale, that it is to direct the traffic of the own node to the other nodes to conform the *Willingness* appropriately. It was observed that way that the network grows is interesting to leave the parameter *Willingness* to direct the whole traffic always.

Already in the AODV protocol the parameters that were modified are: grat-route-reply-flag (*true* for small scale, *false* for medium and large scale), dest-only-flag (*true* for small scale, *false* for medium scale and *true* for large scale), ackrequired (*true* for small scale, *true* for medium scale and *false* for large scale), local repair(*true* for small scale, *true* for medium scale and *false* for large scale). Already the parameters ttl’s were maintained the same ones as the original values. These changes in those parameters contributed to privilege some moments in the network, for instance, the parameter ack-required, in this parameter, the need is verified to guarantee a return of an ACK (*acknowledgment*) only in the small and medium scales, because in the large scale if it doesn’t turn necessary the sending of an ACK, because in this case it can get a great accumulation of messages of ACK in the network. This same concept of the ack-required was used for other above mentioned parameters.

It is valid to stand out that in the Tables 2 and 3, the self-optimization is made in way *off-line*, in other words, the data are externally optimized by MATLAB and later coupled inside the self-optimization module that if found implemented inside the simulator OPNET.

### 4.4. Simulation

This section describes the equipment and the simulation scenario used for the analysis of the acting among the protocols OLSR and AODV pattern and OLSR and self-optimized AODV and configured. A computer M105 Toshiba with 1.66 GHz Duo T2300, 1.5 GB DDR2, 100 GB with Windows XP to accomplish the simulations. Already the simulated scenario is limited to a network in WMN intra-campus that uses the pattern 802.11 g for mesh communication and also the method of communication only-radios, in other words, a single plate of connected network to the node gateway, which serves so much to create it backbone among the nodes gateways, as to distribute access to the clients. In this scenario, several comparisons were accomplished with standard values and with values obtained through the self-optimization methods and self-configuration. The metric of comparison was based on the metric ones, global and specific places, described in the analysis section.

**Figure 5.**
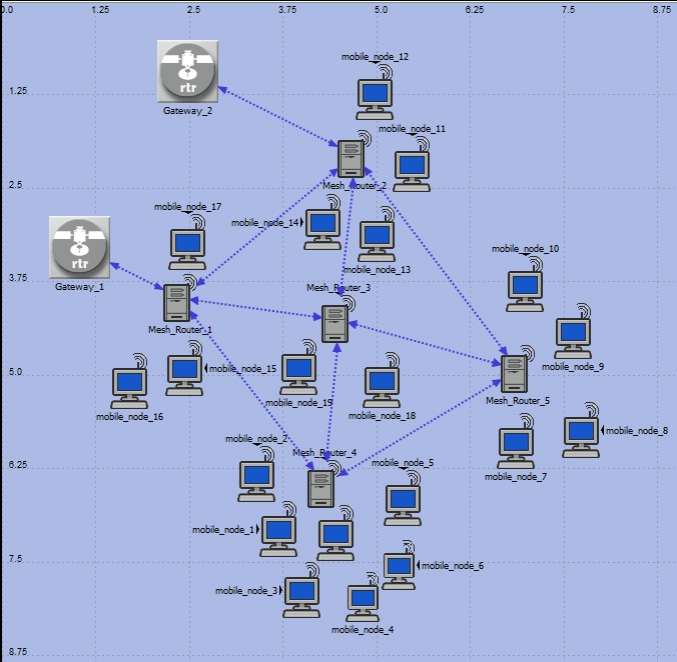
Example of the scenario intra-campus used for simulation of the nodes disposed in an area of 2,000 × 2,000 m^2^

The Figure 5 illustrates the scenario of WMN intra-campus. The scenario consist an area of 2,000 × 2,000 m^2^ and it presents an amount of mobile and fixed devices that they communicate. That communication is made by using multiple-hops through a single network interface mesh, in the pattern 802.11 g *(Direct Sequence)*, with data rate of 11 Mbps. That network type is also known as network in WMN only-radios. The same scenario was used to simulate the protocols OLSR and self-organized AODV being compared with the approach pattern. The growth of the network is configured by the nodes clients’ entrance inside the network, in other words, the measure that the nodes clients enter in the network is counted through the agent of control of the size (NS_Agent) the network’s size.

To accomplish the experiments with the self-optimization methods and self-configuration coupled in the protocols OLSR and AODV, the simulator OPNET version 11.5 was used, with the module for networks without activated thread. The Table 4 presents the used parameters. In the simulations the propagation model was used *Two Ray Ground* that simulates the physical phenomena: it forces, interference and delay in the propagation of the sign, abstracting the real features of WMN. The pattern 802.11g was used to be the variant base of our experiments. For the model of traffic, different demands acted by the stippled arrows were configured for the proposed scenario, such as: HTTP, FTP, VOIP and also the real traffic captured by the tool MRTG. This last one was imported by the simulator through the function Import Baseline Loads. THE antenna type used was the unidirectional, because as already mentioned, each node has a covering area of some meters and it communicates through multihops.

The mobile-nodes clients, move respecting the limits of the propagation of the used sign, that in this case used *Random Waypoint* [35], and the course of each mobile node is characterized as a zigzag. The pattern of speed of the nodes mobile clients was defined as 1 and 2 m/s. That speed imitates a person’s behavior walking in a random way among the propagation limit of the proposed scenario, that it is of 2,000 × 2,000 *m*^2^. The time of simulation was of 60 minutes and the simulations were repeated five times, leaving his/her average to guarantee the largest reliability of the results.

The parameters that differentiate those configurations are: *BSS identifier* and data rate (bps). THE first should be used to identify which gateway will be associated by the customer. Already the second, it has a larger data rate, of 2 Mbps, while the customer uses 1 Mbps. The other parameters are the same for both nodes. It is interesting to emphasize that the customer associates to a node gateway according to the covering area of the same. When there is a node customer’s mobility, this associates to the node closer gateway. Each node gateway has a single number of association.

## 5. Analysis and Results

Next are presented the results obtained through the comparison among protocols OLSR and AODV default jointly OLSR and AODV Self-organized, according to the above described metric.

(3) $$\mathrm{Overhead}=\frac{\mathrm{Total}\_\mathrm{Menssages}\_\mathrm{HELLO}\_\mathrm{or}\_\mathrm{Menssages}\_\mathrm{TC}}{\mathrm{Total}\_\mathrm{of}\_\mathrm{packets}\_\mathrm{in}\_\mathrm{Network}}*100$$

**Figure 6.**
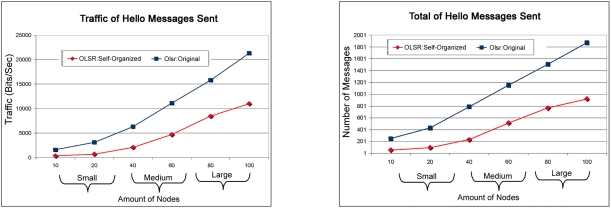
Traffic of HELLO messages sent between OLSR:Self-Organized and OLSR:Original versus Total of HELLO messages sent between OLSR:Self-Organized and OLSR:Original.

In Figure 6 is presented a comparison between OLSR: Self-Organized and OLSR: Original in terms of traffic and total of HELLO messages sent. We can see that when the number of nodes increases into network, all differences between the two protocols that begins to be stronger. For this reason we can conclude that when the network grows in number of nodes clients the OLSR: Self-organized protocol inject less messages of HELLO into the network.

**Figure 7.**
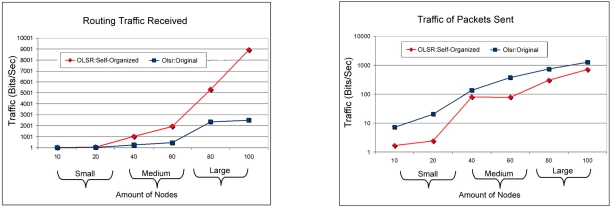
Relationship of the traffic of packages received between the OLSR: Self-organized protocol and OLSR: Original versus Relationship of the traffic of packages sent between the OLSR: Self-organized protocol and OLSR: Original.

Figure 7 shows the comparison of routing traffic sent and received between OLSR:Self-Organized and OLSR:Original. Thus, it is observed that the OLSR:Self-Organized obtained better performance due to the fact that it received more packages and sent less maintenance and control messages in the network. In addition, the OLSR:Self-Organized receive more packages because it has less dropped packages according to Figure 9.

It can still be observed that in the small scale the same behavior of both comparisons, but already in the scales average for big the OLSR: Self-organized received more packages than OLSR: Original. While the most specific analysis of the total number of HELLO messages sent as having shown in the Figure 6, it can be observed that in the small medium and large scales injected a smaller number of messages HELLO in the network. It is also observed for the medium scale a very slight curve of packages for the protocol OLSR:Self-organized, this happened due to the adjustment of the parameter of TC_Interval for 0.0588 in the medium scale as observed in the Table 2. This value was originated from the specific analysis of the parameter using the optimization function.

**Figure 8.**
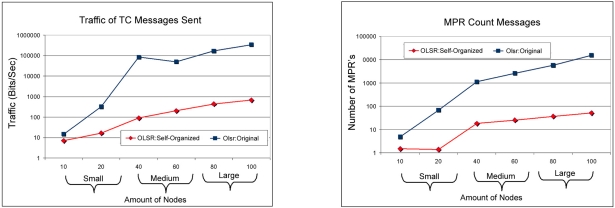
Comparison of the traffic of TC messages between of OLSR: Self-organized protocol and OLSR: Original versus Comparison of the number of MPR messages between OLSR: Self-organized protocol and OLSR: Original.

**Figure 9.**
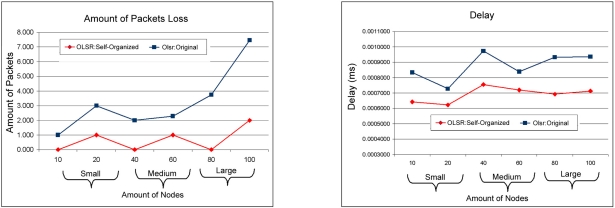
Comparison of the amount of dropped packages between OLSR: Self-organized protocol and OLSR: Original versus Relationship of the end-to-end delay among OLSR: Self-organized and OLSR: Original.

Figure 8 shows that the comparison among TC (topology Control) messages and the number of MPR messages between the protocol OLSR: Self-Organized and the OLSR: Original. In this point of view the OLSR: Self-organized injected less control and maintenance of routes messages. Consequently, injecting less messages in the network avoids overload and excessive accumulation of messages, because it is a feature of the protocol OLSR to have a high overload due to technique used to change messages in the network. Finally the OLSR: Self-organized protocol obtained better performance than OLSR: Original in almost all metrics analyzed. In Figure 9 it is possible to see the amount of discarded packages for the protocols during the simulation. In this point of view it is observed that the OLSR: Self-organized protocol obtained a smaller discard of packages in relation to the protocol OLSR: Original in all the scales of network and it is also observed that when the networks grow in amount of devices we can see that discard for the protocol OLSR:Original tends to be larger. It can observed that in 10, 40 and 80 nodes the discard was zero in relation to packages in bits/second. We can see too the end-to-end delay among OLSR: Self-organized and OLSR: Original and the OLSR: Self-Organized obtained smaller delay in comparison with OLSR:Original.

**Figure 10.**
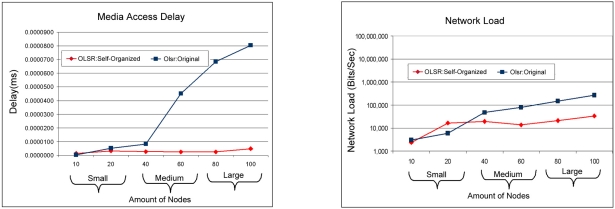
Comparison of the access delay to the middle between the OLSR: Self-organized protocol and OLSR: Original versus Relationship of the load of the network among of the OLSR: Self-organized protocol and OLSR: Original.

In Figure 10 it is possible to see the access delay to the middle of the OLSR: Self-organized protocol that is smaller in relation to OLSR:Original, and it can also be observed that the way the amount of nodes grows in the network, in other words, of the medium scale for big scale, the approach of self-optimization has a more significant acting. Consequently the network load can be visualized, that as observed the OLSR: Self-organized protocol when in the small scale it obtains a larger load, while the protocol OLSR:Original obtains a smaller load. But in compensation in the scales average for big the OLSR: Self-organized reduced the load of the network. This fact is explained due to the effort of the protocol OLSR to find the destiny routes, like him it is used of flood by MRP’s, in other words, it is needed an effort to find the routes, that effort is compensating when the network grows in size, passing of the scales average to large. Confirming that the protocol OLSR doesn’t adapt very well in networks of small load.

### 5.1. Capacities of Self-optimization and Self-configuration Applied into AODV protocol

**Figure 11.**
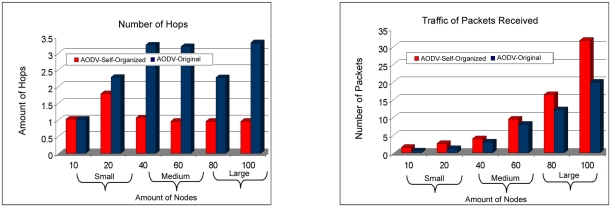
Comparison of the number of hops for route between the AODV: Self-organized and AODV: Original versus Comparison of the traffic of packages received among AODV: Self-organized and AODV: Original.

In Figure 11 the relationship of the number of hops is observed by route of the AODV: Self-organized protocol, in which it is possible to verify that the same needed a smaller number of hops to find the node destination in large scale. Otherwise, in small scale the number of hops was practically the same as the protocol AODV: Original.

**Figure 12.**
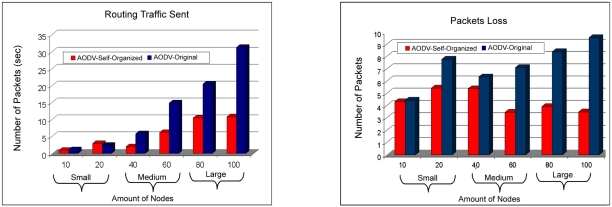
Comparison of the traffic of packages sent among AODV: Self-organized and AODV: Original versus Comparison of the number of dropped packages of the AODV: Self-organized protocol and AODV: Original.

The number of packages received by the AODV: Self-organized protocol was larger in relation to the protocol AODV:Original. That improvement was provided because of the less packet loss or collisions in the network and also for the adjustment of the packages that they were in *buffer*, waiting for a route answer for a route request. Now in Figure 12 the relationship of sent packages can be observed, in which the AODV: Self-organized protocol obtained a smaller amount of packages sent in all the scales.

It is valid to stand out in this case that the amount of sent packages is a metric one that it counts all the packages, besides the control packages and maintenance of routes, it is observed then that the AODV:Self-organized protocol sent a smaller number of control packages and maintenance of routes in this experiment. It is observed that the AODV: Self-organized protocol obtained smaller loss of packages in all the scales. But it is interesting to point out that the way the network grows in size, or be amount of devices clients, and it reaches the scales average and big, the loss of packages of AODV:Original is larger in comparison to the AODV: Self-organized. That reinforces that the way network grows in size there is a smaller proportionate loss of packages for the AODV: Self-organized protocol.

**Figure 13.**
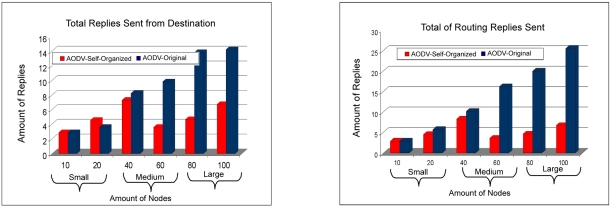
Comparison of total number of replicas sent to destination nodes of protocol AODV: Self-organized e AODV:Original versus Comparison of total replicas of routing sent between protocol AODV: Self-Organized and AODV:Original.

Already in Figure 13 the total number of replicas can be visualized sent to the destiny nodes and also the routes replies, which the AODV: Self-organized protocol needed to send a smaller number of routing replies.

**Figure 14.**
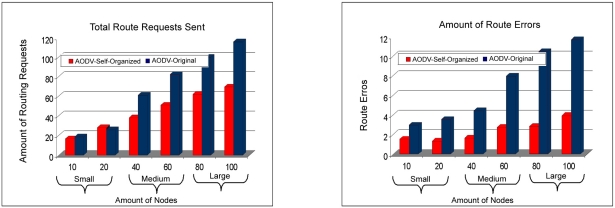
Comparison of total requests of routes sent by AODV: Self-Organized e AODV: Original versus Comparison of total of routes errors between AODV: Self-Organized and AODV: Original.

**Figure 15.**
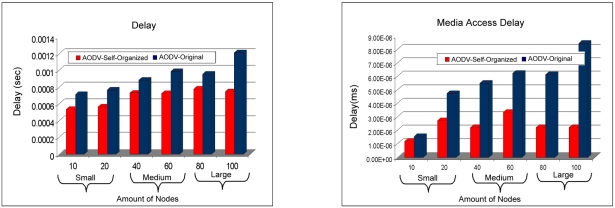
Comparison of the delay of the AODV: Self-organized protocol and AODV: Original versus Comparison of the access delay to the middle of the AODV: Self-organized Protocol and AODV: Original.

In Figure 14 the total of requests of routes can be visualized sent together with the total of mistakes of routes. It can be observed that the AODV: Self-organized protocol needed to send a smaller number of requests of routes, compared to the protocol AODV: Original. With what the network wins in stability, or be the AODV: Self-organized protocol needed of a smaller number of requests of routes for each neighboring node. Finally, it is noticed a smaller number of error routes of the AODV: Self-organized protocol in all the scales of the wireless mesh network (small to large).The Figure 15 presents the delay among the Protocols AODV: Self-organized and AODV: Original. It is observed that the AODV: Self-organized protocol obtained a smaller delay of discovery of broken in all the scales. In this sense, is also observed the access delay to the middle, that once again the AODV: Self-organized protocol obtained better performance in all the scales in comparison with AODV Originally proposed.

**Figure 16.**
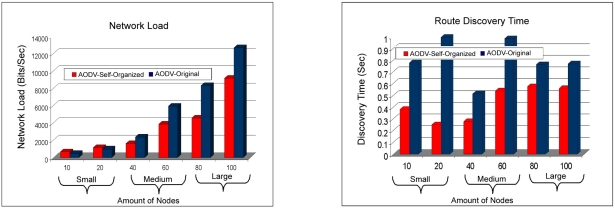
Comparison of the load of the network among AODV: Self-organized and AODV: Original versus Comparison of the time of route discovery among AODV: Self-organized and AODV:Original.

The Figure 16 exhibit the load of the network and the time of route discovery. In this case it is observed that the load of the network of the AODV: Self-organized protocol is smaller than AODV:Original in the scales average for large. While in the small scale the protocol AODV:Original has a better performance. That is explained due to the fact that the protocol AODV originally proposed to use technique for demand, in atmospheres that the scale of the network is small, in other words, the amount of devices is small in the network, the protocol needs to locate their destiny nodes through the demand technique, that increases the number of requests of routes also increasing in consequence the load of the network. The time of route discovery is visualized, in which the AODV: Self-organized protocol obtained better time of route discovery in all the scales of the WMN.

**Figure 17.**
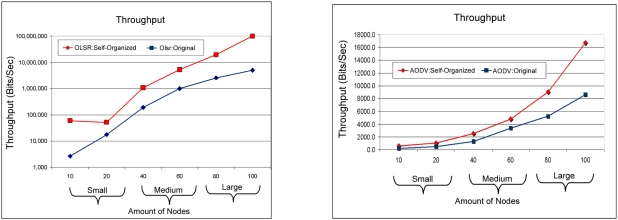
Comparison of throughput between the OLSR:Self-organized protocol and OLSR:Original versus AODV:Self-organized protocol and AODV:Original.

Figure 17 symbolizes the relationship of the throughput of network, among OLSR: Self-organized compared to OLSR: Original. It can be visualized in this Figure that the way the nodes enter in the network, together with the variation of the small scale for medium scale until the big scale, it is noticed a significant improvement of the throughput of the OLSR: Self-organized protocol.

The comparison of the throughput can be observed in Figure 17. In this case the AODV: Self-organized obtained a significant throughput improvement when the scale of the network is growing. While in the small scale a minor difference is observed.

After the OLSR and AODV results we argue that this specific analysis on protocols is better for sceneries where the position of the nodes is practically known, due to techniques of finding the routes for the general knowledge of the network, this process demand a very big load of control messages and maintenance of routes. It was observed that the self-adjustment of these intervals can guarantee a good acting of protocols when the number of nodes growing to medium for large scales.

### 5.2. Comparison between OLSR:Self-organized and AODV:Self-organized

Besides the specific analyses of each in accordance protocol seen in the previous sections, the comparison among the protocols OLSR and AODV was also analyzed after applying the capacities of self-configuration and self-optimization.

In Figure 19, it is observed that applying the self-optimization technique hears a significant improvement in the network throughput, due to the self-adjustment of the parameters it adjusts to the size of the network, in comparison with the protocol originally proposed. It can also observe that in the small scale the protocol OLSR obtained a better throughput, while in the scales average for large AODV: Self-organized protocol obtained better throughput. This fact can be explained due to the features of each protocol, for instance. The protocol AODV is used by demand to find the routes for the destiny nodes, while the protocol OLSR has a table of broken pre-updated with the routes for the destiny nodes.

By analyzing scales it is observed that the way scale increases in size, or be amount of devices clients in the network, the protocol OLSR creates a larger overload affecting like this the throughput of the whole network, while the ADOV: Self-optimized protocol obtains the routes according to the demand avoiding like this that overload the throughput and increasing it in consequence. In this case it is concluded that AODV: Self-organized protocol obtains better throughput when the scale of the network goes from the existing average to the large one.

**Figure 18.**
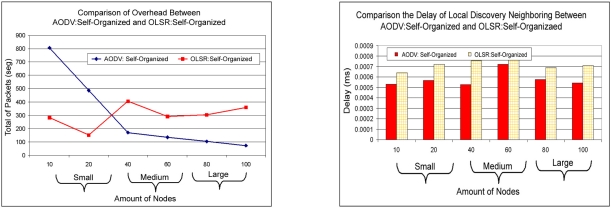
Relationship of the overload between OLSR: Self-organized and AODV:Self-organized versus Relationship of the delay of route discovery between OLSR: Self-organized and AODV:Self-organized.

**Figure 19.**
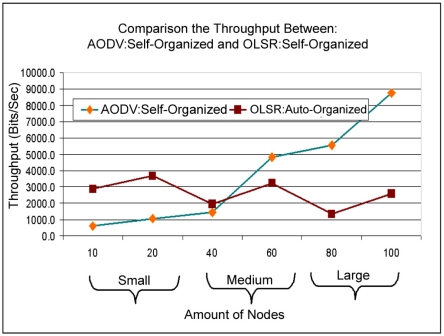
Relationship of the throughput between OLSR: Self-organized and AODV: Self-organized.

The delay of route discovery is measured in Figure 18, it can be observed that the AODV: Self-organized protocol obtained a smaller delay of discovery of broken in the small scales, medium and large. This result is interesting because, due to the protocol AODV the use of technique of demand, in consequence that technique creates a certain delay to find the route for the destiny node. It is concluded here in this experiment the AODV: Self-organized protocol obtained a smaller delay in comparison to OLSR: Self-organized.

The overload of the network is analyzed in Figure 18, in which can be observed that the AODV: Self-organized protocol obtains larger overload to find the routes in the small scale, while the OLSR: Self-organized protocol has a smaller overload in the small scale, but when the scale of the network in mesh changes the average into a large one. It is observed that the overload of packages of the AODV: Self-organized protocol decreases and the one of the OLSR: Self-organized increases. It is concluded here in this experiment that the AODV: Self-organized protocol adapts better in WMN of medium and large load in comparison to OLSR: Self-organized.

In this work the results and simulations were presented to validate the proposed architecture. Initially we simulated their capacity of self-optimization and self-configuration applied to the routing protocols OLSR and AODV. As result, a significant improvement was obtained in the acting of those applied protocols in the wireless mesh networks, of small, medium and large load. The metric ones proposed for analysis of that acting showed in first instance that the self-configuration capacities and self-optimization applied to the mentioned protocols, improve the acting of the WMN in comparison to original approach, in which uses the standard parameters of the protocols. Finally, as analysis of comparisons among the protocols OLSR: Self-organized with the AODV: Self-organized, a better use of the AODV: Self-organized protocol is observed in atmospheres in that the WMN alternates among the scales average and large ones.

## 6. Conclusions

In this work an architecture was proposed for self-organization in WMN using as base the protocols OLSR and AODV, together with the software agents’ technology, to aid the self-organization capacities (self-optimization and self-configuration) in the adjustment of the values of the protocol’s parameters of foregoing routing. For such, the division of the size of the network was proposed in scales.

It was noticed that when revising the specialized literature, that few works focusing the subject of the self-organization for networks in WMN still exist. The architecture proposed still involves the definition of concepts originating from of the autonomous computation, integrated into the software agents’ technology.

The algorithm that verifies the scale of WMN was coupled in the capacity of self-configuration, while the capacity of self-optimization obtains an optimized value and this was put inside of the five parameters of the protocols OLSR (Hello-Interval, Tc-Interval, Neighbor-Hold-time, Tc-Hold-time, Duplicate-Hold-time) and AODV (Route-Request-Retries, Hello-Interval, Active-Route-Timeout, Allowed-Hello-Loss, Net-Diameter). Utilized no linear optimization without restrictions to adapt the objective hyperbolic and cotangent function to the analysis behavior of the interval variation of the mentioned parameters. That optimized value was directly added in those parameters according to the scale in that the network was in a certain moment. Being compared the self-optimization capacities and self-configuration implemented in this dissertation, to the works of [19,36,37]. it can be concluded that the implemented capacities obtained better performance in WMN. Beyond that we analyzed five parameters of each routing protocol, while, the works of [36,37] analyzed the HELLO messages rate in the network that is configured through the parameter Hello-Interval. Already the work of [19] analyzed only the self-configuration capacity and self-optimization in networks MANET, and not in WMN.

For the scales it was chosen to divide the WMN in three sizes: small, medium and large. The size of the network was obtained through the agent of denominated software NS-Agent, that is responsible for the process of collection of the size of the network and later, for sending that information to other nodes gateways. It was observed that the capacities of self-optimization and self-configuration proposed in this work has strong correlation, because one is complement of the other: while one optimizes, the other configures the values in the parameters of the mentioned protocols.

With all the implemented capacities, it is expected that the WMN can have:

- Larger acting, being applied the capacities of self-optimization and self-configuration;
- High tolerance to fail, being applied the self-cure capacity;
- More safety to attack and intrusions, being applied the self-protection capacity.

In this paper the acting was emphasized with the implementation of the capacities of self-optimization and self-configuration, with agents NS-Agent’s implementation.

Verifying the results of the simulations a significant improvement of the acting of the network was observed in WMN, using the capacities of self-optimization and self-configuration coupled in the routing protocols OLSR and AODV.

The simulations still showed specific results in the protocols OLSR: Self-organized and AODV: Self-organized, among which can be mentioned.

- Better in the throughput of the WMN;
- Better in the delivery of the packages, because it was noticed a smaller loss of packages;
- Smaller delay of the discovery of the routes among the neighboring nodes;
- Smaller injection of control messages and maintenance in the network.

It could also be verified that the AODV: Self-organized protocol obtained better acting compared to the OLSR: Self-organized protocol, while the scale of the WMN changed average into a greater load.

It is been worth to emphasize, that the proposed architecture is still in development phase, lacking to couple the self-cure capacities and self-protection together with the agent NM-Agent. Efforts should be made so that hereafter the architecture can become a reference in self-organization for networks in WMN.

Finally, every context of this work made an including learning in several aspects possible regarding the networks in WMN and to the self-organization capacities (self-optimization, self-configuration, self-cure and self-protection), allowing the acquired knowledge to be reviewed for the project and conception of the proposed architecture.

## Figures and Tables

**Table 1.** Example of parameter hello-interval using the throughput as metric of analysis.

**Nodes**	0.5	1	1.5	2	...	40	50
**SMALL (10)**	28.41469	24.5938	24.59264	71.20893		16.16489	16.16489
**SMALL (20)**	1290.703	861.9226	656.1949	575.5491		248.0969	248.0969
**SMALL (30)**	659.5588	443.2582	340.3938	323.379		132.1309	132.1309
**MEDIUM(40)**	2646.458	1704.578	1303.33	1163.118		765.0526	765.0526
**MEDIUM(60)**	6692.721	3773.002	2860.065	2285.047		882.2232	837.5746
**MEDIUM(70)**	4669.589	2738.79	2081.698	1724.083		823.6379	801.3136
**LARGE(80)**	8583.169	5175.946	3907.206	3290.569		1620.508	1664.479
**LARGE(90)**	11324.1	6876.763	5405.711	4612.651		2375.319	2165.133
**LARGE(100)**	9953.633	6026.355	4656.459	3951.61		1997.914	1914.806

**Table 2.** Description of OLSR protocol parameters after of self-optimization process.

OLSR Parameters	Standard	Small	Medium	Large
node_willingness	default	medium	high	always
**hello_interval**	2,0	8.7311	2.9802	2.8312
**tc_interval**	5	1.3388	0.0588	9.8348
**neighbor_hold_time**	6	2.3283	1.6764	1.4107
**tc_hold_time**	15	4.0745	3.7253	2.6822
**dup_hold_time**	30	20	35	10

**Table 3.** Parameters description of the protocol AODV after the Self-Optimization process.

AODV Parameters	Standard	Small	Medium	Large
**route_request_retries**	1	8.2171	3.2413	2.9104
route_request_rate	10	10	10	10
grat_route_reply_flag	False	True	False	False
dest_only_flag	False	True	False	True
ack_required	False	True	True	False
**hello_interval**	1,0	3.2906	5.9951	9.0137
**active_route_timeout**	3,0	2.7784	4.3656	5.8208
**allowed_hello_loss**	2	7.0288	2.5524	1.3097
**net_diameter**	35	8	3	1
node_traversal_time	0,04	0,04	0,04	0,04
route_error_rate	10	5	10	15
timeout_buffer	2	2	2	2
pkt_queue_size	−1	−1	−1	−1
local_repair	True	True	True	false
ttl_start	1	1	1	1
ttl_increment	2	2	2	2
ttl_threshold	7	7	7	7
local_add_ttl	2	2	2	2

**Table 4.** Description of the simulation parameters used in the scenario intra-campus.

Scenario:	Net Intra-campus of 2000 x 2000m2
Nodes clients:	Fixed and Pieces of furniture with different mobilities (1, 2 m/s)
Channel:	Single Radio
Model of Propagation:	Two Ray Ground
Type of Interface:	Wireless NIC
Layer PHY:	802.11g
Model of the Antenna:	Omni-Antenna
Model of Mobility:	Random Waypoint
Protocol of Transport:	TCP AND UDP
Amount of Gateways in Static Mesh:	5
Size of the net:	Scales small, medium, large
Model of traffic:	HTTP, FTP, VOIP, REAL
Width of Band of the Nodes:	2 Mbps
Routing protocols:	AODV AND OLSR
Type of the Layer of Connection:	LL
Type of Line:	FIFO
Maximum of Packages in Line:	50
Time of Simulation:	60 minutes
Trust interval	95%
